# Creatine Loading Does Not Preserve Muscle Mass or Strength During Leg Immobilization in Healthy, Young Males: A Randomized Controlled Trial

**DOI:** 10.1007/s40279-016-0670-2

**Published:** 2017-01-05

**Authors:** Evelien M. P. Backx, Roland Hangelbroek, Tim Snijders, Marie-Louise Verscheijden, Lex B. Verdijk, Lisette C. P. G. M. de Groot, Luc J. C. van Loon

**Affiliations:** 1 Department of Human Nutrition, Wageningen University, Wageningen, The Netherlands; 2 Top Institute Food and Nutrition, Wageningen, The Netherlands; 3 NUTRIM School of Nutrition and Translational Research in Metabolism, Maastricht University Medical Centre, 6200 MD Maastricht, The Netherlands; 4 Department of Surgery, Maastricht University Medical Centre+, Maastricht, The Netherlands

## Abstract

**Background:**

A short period of leg immobilization leads to rapid loss of muscle mass and strength. Creatine supplementation has been shown to increase lean body mass in active individuals and can be used to augment gains in muscle mass and strength during prolonged resistance-type exercise training.

**Objective:**

Our objective was to investigate whether creatine loading can attenuate the loss of muscle mass and strength during short-term leg immobilization.

**Methods:**

Healthy young men (*n* = 30; aged 23 ± 1 years; body mass index [BMI] 23.3 ± 0.5 kg/m^−2^) were randomly assigned to either a creatine or a placebo group. Subjects received placebo or creatine supplements (20 g/d) for 5 days before one leg was immobilized by means of a full-leg cast for 7 days. Muscle biopsies were taken before creatine loading, prior to and immediately after leg immobilization, and after 7 days of subsequent recovery. Quadriceps cross-sectional area (CSA) (computed tomography [CT] scan) and leg muscle strength (one-repetition maximum [1-RM] knee extension) were assessed before and immediately after immobilization and after 1 week of recovery. Data were analyzed using repeated measures analysis of variance (ANOVA). Data are presented consistently as mean ± standard error of the mean (SEM).

**Results:**

There was a significant overall increase in muscle total creatine content following the 5-day loading phase (*p* = 0.049), which appeared driven by an increase in the creatine group (from 90 ± 9 to 107 ± 4 mmol/kg^−1^ dry muscle) with no apparent change in the placebo group (from 88 ± 4 to 90 ± 3 mmol/kg^−1^; *p* = 0.066 for time × treatment interaction). Quadriceps muscle CSA had declined by 465 ± 59 and 425 ± 69 mm^2^ (*p* < 0.01) in the creatine and placebo group, respectively, with no differences between groups (*p* = 0.76). Leg muscle strength decreased from 56 ± 4 to 53 ± 4 kg in the creatine and from 59 ± 3 to 53 ± 3 kg in the placebo group, with no differences between groups (*p* = 0.20). Muscle fiber size did not change significantly over time in either group (*p* > 0.05). When non-responders to creatine loading were excluded (*n* = 6), responders (*n* = 8; total creatine content increasing from 70 to 106 mmol/kg^−1^) showed similar findings, with no signs of preservation of muscle mass or strength during immobilization. During the subsequent recovery phase, no differences in muscle mass or strength were found between the two groups (*p* > 0.05).

**Conclusion:**

Creatine supplementation prior to and during leg immobilization does not prevent or attenuate the loss of muscle mass or strength during short-term muscle disuse.

***NIH Clinical Trial Registration Number:*** NCT01894737 (http://www.clinicaltrials.gov/).

## Key Points

**Table:** 

Leg immobilization for 7 days led to a 5.5% reduction in leg muscle mass and a 7.6% decline in muscle strength.
Creatine supplementation prior to and during leg immobilization did not attenuate the decline in leg muscle mass or strength.
Creatine supplementation did not prevent disuse atrophy.

## Background

Recovery from injury or illness often requires a period of inactivity. Even short periods of disuse lead to a substantial loss of skeletal muscle mass and strength [1, 2]. The loss of muscle mass and strength is associated with a longer recovery period and with a higher risk of injury reoccurrence [3, 4]. Moreover, periods of disuse result in reduced insulin sensitivity [5, 6], a decline in basal metabolic rate [7], and an increase in body fat mass [8]. These factors impair physical functioning and decline metabolic health, causing even more health concerns. Therefore, strategies are warranted to prevent the loss of muscle mass and/or strength during short periods of disuse due to injury or hospitalization.

During the 1990s, creatine supplements became a popular ergogenic aid for athletes. Creatine monohydrate supplementation can increase the phosphocreatine/creatine ratio in skeletal muscle tissue, thereby increasing the capacity for rapid adenosine triphosphate (ATP) resynthesis during repeated high-intensity exercise tasks [9–11]. In accordance, creatine supplementation was shown to effectively augment athletic performance during various exercise tasks characterized by repeated high-intensity exercise and has since become common practice among many athletes [12–14]. Besides increasing high-intensity exercise performance, creatine supplementation has also been reported to increase muscle mass and strength in the presence as well as in the absence of prolonged resistance-type exercise training [9, 15, 16]. The increase in lean mass following creatine supplementation has, at least partly, been attributed to water retention in muscle tissue [11, 17]. The greater osmotic pressure following the increase in creatine content has been suggested to result in muscle cell swelling, which is considered a key stimulus for cell growth [11, 18].

Consequently, it has been hypothesized that creatine loading prior to immobilization may help to prevent muscle loss during a short period of disuse [19]. So far, only two studies have assessed the clinical benefits of creatine supplementation during immobilization to prevent or attenuate disuse atrophy [20, 21]. Whereas creatine supplementation attenuated muscle loss during a 1-week arm immobilization [20], no effect was observed during a 2-week leg immobilization period [21]. Since neither study included a creatine-loading phase to allow an increase in muscle creatine content before immobilization [20, 21], it remains to be determined whether creatine loading prior to (leg) immobilization can preserve muscle mass and strength in males.

We hypothesized that creatine loading would prevent or attenuate the loss of muscle mass and strength during 1 week of leg immobilization. To test our hypothesis, we selected 30 young males to participate in a study in which they were subjected to 7 days of single-leg immobilization. Subjects received either placebo or creatine supplements from 5 days prior to immobilization until the end of the study. To assess the effect of creatine loading, muscle biopsies were taken at baseline and after 5 days of creatine loading (20 g/d). Before and after 7 days of immobilization as well as after 7 days of subsequent recovery, we assessed muscle mass and strength. In addition, muscle biopsies were taken to assess muscle fiber characteristics.

## Methods

### Subjects

After screening, 30 healthy young men (aged 23 ± 1 year; body mass index [BMI] 23.3 ± 0.5 kg/m^−2^) were included in the study. Exclusion criteria included a (family) history of thrombosis; any back, leg, knee, or shoulder complaints that could interfere with the use of crutches; and any co-morbidities interacting with mobility or muscle metabolism of the lower limbs (e.g., arthritis, spasticity/rigidity, all neurological disorders and paralysis). None of the subjects reported having performed progressive resistance-type exercise training or having used creatine supplements, anticoagulants, corticosteroids, growth hormones, testosterone, immunosuppressants, or exogenous insulin over the previous 6 months. During the screening, subjects gave written informed consent after they were fully informed about the nature and possible risks of the experimental procedures. The study was approved by the Medical Ethics Committee of the Maastricht University Medical Centre and performed in accordance with the Declaration of Helsinki.

### Experimental Design

**Fig. 1:**
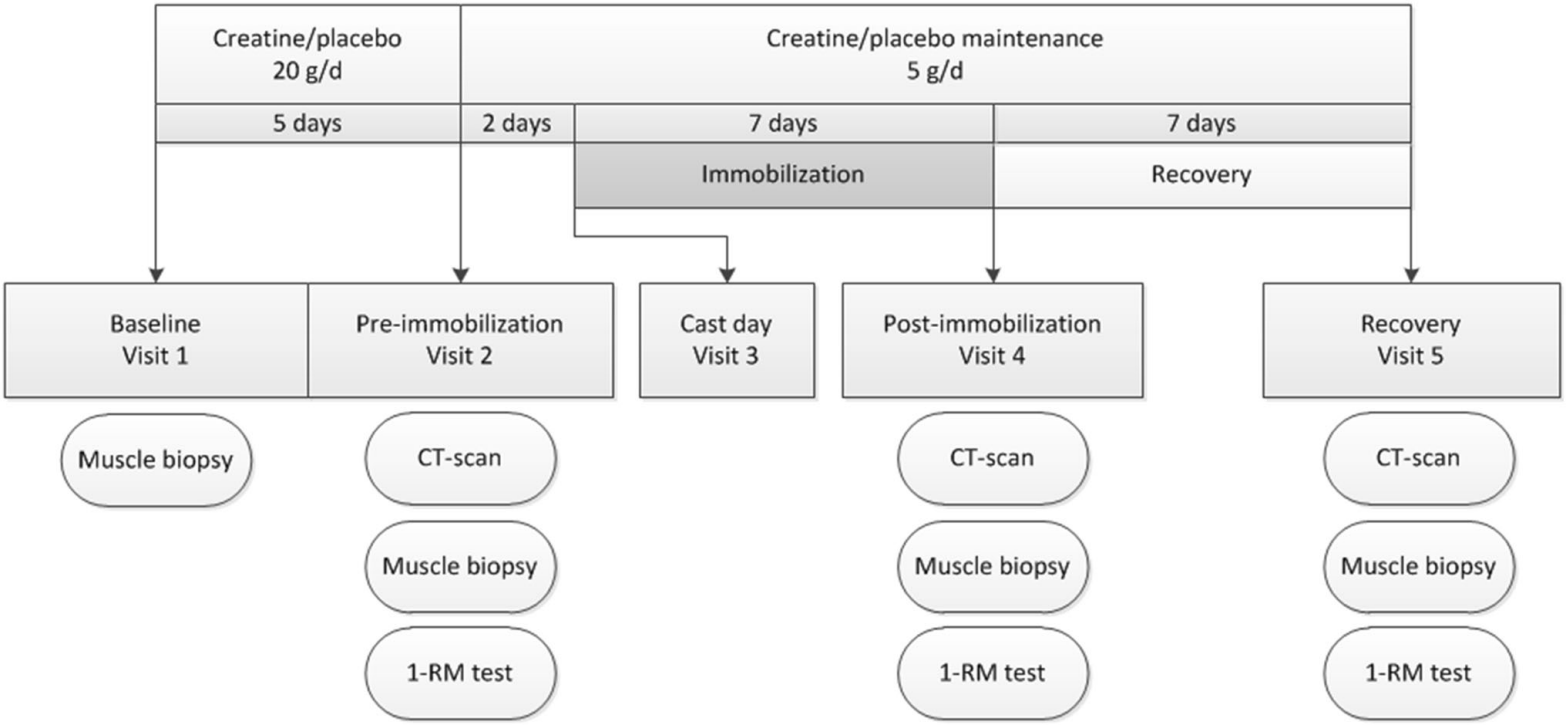
Schematic overview of the study design. *1-RM* one-repetition maximum, *CT* computed tomography

The study was a double-blind placebo-controlled intervention trial with 30 subjects randomly allocated to receive either placebo (*n* = 15) or the creatine supplementation (*n* = 15) (Fig. 1). All subjects reported to the laboratory following an overnight fast on four occasions. Standardized meals (2.9 MJ, providing 51 energy percent [En %] as carbohydrate, 32 En % as fat, and 17 En % as protein) were consumed the evening before all 4 test days. On the first test visit (baseline), body weight was assessed and a muscle biopsy sample was taken from the vastus lateralis muscle. Subjects started with the creatine or placebo supplements directly after the baseline test. Subjects came back in 5 days after the baseline test for the pre-immobilization test. During the pre-immobilization test, a second muscle biopsy was taken to assess the effect of creatine loading on muscle creatine content and a baseline assessment of muscle mass (computed tomography [CT] scan upper legs) was conducted. Muscle strength was estimated using a one-repetition maximum (1-RM) knee extension test 1 h after the biopsy; 2 days after the pre-immobilization test, one leg was immobilized by means of a full leg cast for 7 subsequent days. On the day of the cast removal, and after 7 days of recovery, another muscle biopsy was taken and muscle mass and strength assessments were repeated.

### Measurements

Body weight was measured with a digital balance with an accuracy of 0.1 kg (SECA GmbH, Hamburg, Germany). Muscle biopsies were taken from the middle region of the vastus lateralis muscle under local anaesthesia, ~15 cm above the patella and ~3 cm below entry through the fascia, using the percutaneous needle biopsy technique [22]. All biopsies were taken from the leg that was subsequently immobilized. Muscle samples were dissected carefully, freed from any visible non-muscle material, and immediately frozen in liquid nitrogen (biochemical analyses) or liquid nitrogen-cooled isopentane (histochemical analyses). Muscle samples were stored at –80 °C until further analysis.

Muscle mass was assessed with a single-slice CT scan (Philips Brilliance 64, Philips Medical Systems, Best, the Netherlands) to assess the cross-sectional area (CSA) of the quadriceps muscle. The scanning characteristics were as follows: 120 kV, 300 mA, rotation time 0.75 s, and field of view 500 mm. With subjects lying supine with their legs extended and feet secured, a 3-mm axial image was taken 15 cm proximal to the top of the patella. The precise scan position was marked with semi-permanent ink for the duration of the intervention period to ensure accurate repeated measurements. For analysis, muscle area of the legs was selected between –29 and 150 Hounsfield units [23], after which the quadriceps muscle was selected by manual tracing using ImageJ software (version 1.49p, National Institute of Health, Maryland, USA) [24–26]. To estimate muscle strength, 1-RM measurements were performed [5] for each leg individually on a knee extension machine (Technogym, Rotterdam, The Netherlands). In short, during the screening visit, the lifting technique was demonstrated, and a familiarization trial was performed to ensure proper execution of the exercise protocol. Using the multiple-repetitions testing procedure [28], 1-RM was estimated as described previously [27]. This estimate was then used during the following visits to determine the initial load for the actual 1-RM test. During the actual 1-RM test, subjects warmed up and then performed a single repetition with the load set at ~90% of the estimated 1-RM. Load was then increased by 2.5–5.0% after each successful lift, until failure.

### Supplements

The placebo group received a placebo supplement consisting of maltodextrin 7.5 g and dextrose monohydrate 7.5 g (both from AVEBE, Veendam, the Netherlands). The creatine group received the same supplement with the addition of creatine monohydrate 5 g (Creapure AlzChem, Trostberg, Germany). The creatine supplements could not be distinguished from the placebo supplement with respect to color, taste, smell, or appearance. All subjects were instructed to dissolve the supplement in lukewarm tap water prior to ingestion. During the first 5 days of creatine loading, subjects consumed four supplements per day (providing a total of creatine 20 g/day for the creatine group) at breakfast, lunch, dinner, and immediately before going to bed (approximately 5-h intervals between supplements). Thereafter, subjects continued with one supplement per day as a maintenance dose (containing creatine 5 g per day for the creatine group) during the subsequent immobilization and recovery period. The applied creatine supplementation protocol was designed according to the guidelines on creatine supplementation set by the American College of Sports Medicine [29].

### Limb Immobilization

At 2 days after the pre-immobilization test, subjects attended the Casting Room at Maastricht University Medical Centre at 8.00 a.m. to have a full leg cast fitted to induce knee immobilization. The leg to be immobilized was randomized and counter-balanced between left and right. The circular leg cast extended from 10 cm above the ankle to approximately 25 cm above the patella. The knee was casted at a 30° angle of flexion to prevent subjects from being able to perform any weight-bearing activities on the casted limb. Subjects were provided with crutches for proper ambulation. Throughout the immobilization period, subjects were instructed to perform a series of simple daily ankle exercises (i.e., plantar and dorsal flexion, and circular movements of the entire foot) to keep the calf muscle pump activated in the immobilized leg, thereby minimizing the risk of developing deep vein thrombosis. Prior to the start of the third test visit, subjects visited the casting room to have the cast removed. Following cast removal, subjects were transported by wheelchair to prevent any weight bearing on the immobilized leg until the muscle biopsy sample was collected.

### Diet and Physical Activity

Subjects were asked to maintain habitual dietary intake during the entire intervention period. To check for any deviations, the participants completed dietary intake records, including food sources, estimation of the portion size using household measures, and the timing of intake. Dietary intake records were completed before and during the immobilization period on 2 weekdays and 1 weekend day. DieetInzicht software [7], based on the Dutch food composition table (NEVO table) 2011, was used to analyze dietary intake records. Alcohol intake was allowed until 48 h before every test visit. Subjects refrained from any heavy physical exercise during the entire intervention period. Therefore, a 3-day activity journal was completed by the participants on the same days as the dietary intake records.

### Muscle Total Creatine Analysis

One fraction of the muscle sample (~50 mg wet tissue) was freeze dried and pulverized. The method of muscle creatine and creatine phosphate analyses was based upon the method described by Harris et al. [30]. After adding perchloric acid (250 μl PCA 1 M for 10 mg dry muscle), samples were agitated on ice for at least 10 min, after which they were centrifuged (1000*g*, 10 min, 4 °C). KHCO3 (105 μl, 2 M) was added to 175 μl of acid extract to neutralize the samples. Neutralized samples were centrifuged (1000*g*, 10 min, 4 °C), and supernatants were immediately used for analysis by enzymatic NADH-linked assay to detect changes in NADH concentration by spectrophotometry (Cobas Fara, Roche, Switzerland) at 340 nm in duplicate. Creatine and phosphocreatine standards (0.8, 1.6, and 3.2 mM) were analyzed in each run of 24 samples. Muscle total creatine contents were corrected for the highest ATP value of the same subject to account for variations in sample impurities (both raw and corrected data are presented in Table 2).

### Histochemical Analysis

Part of each muscle biopsy sample was frozen in liquid nitrogen-cooled isopentane. These samples were cut into 5-μm thick cryosections using a cryostat at –20 °C. Muscle biopsies were stained for muscle fiber typing and used to assess muscle fiber type composition and type I and II muscle fiber size as described in detail previously [31]. In short, slides were incubated with primary antibodies directed against myosin heavy chain (MHC)-I (A4.840, dilution 1:25; Developmental Studies Hybridoma Bank, Iowa City, IA, USA) and laminin (polyclonal rabbit antilaminin, dilution 1:50; Sigma, Zwijndrecht, the Netherlands). Goat anti-mouse immunoglobulin M (IgM) AlexaFluor555 and goat anti-rabbit immunoglobulin G (IgG) AlexaFluor647 were applied as secondary antibodies (dilution 1:500 and 1:400; Molecular Probes, Invitrogen, Breda, the Netherlands). Images were captured with a fluorescent microscope and analyzed for muscle CSA and fiber-type distribution using ImageJ software.

### Statistics

All data are expressed as means ± standard error of the mean (SEM). The sample size was based upon a power calculation with an expected difference in the loss of quadriceps CSA of 40% between the placebo and creatine group (respectively, 5 vs. 3% loss of quadriceps CSA) [32]. With a power of 80% and a significance level (*α*) of 0.05, at least 13 participants are required per group. To allow for drop-outs, 30 subjects were included.

An independent *t* test was used to assess differences in baseline characteristics between subjects in the placebo and creatine groups. Our main objective focused on the differences between the placebo and the creatine group during immobilization. We used a two-way repeated measures analysis of variance (ANOVA) with time (pre-immobilization vs. post-immobilization) as the within-subjects factor and treatment (creatine and placebo group) as between-subjects factors to compare changes in quadriceps CSA, muscle strength, and muscle fiber characteristics during immobilization. Thereafter, a two-way repeated measures ANOVA was conducted with time (post-immobilization vs. post-recovery) as within-subjects factor to test the differences between the placebo and the creatine groups during the recovery week. A third repeated-measures ANOVA with all three time points (pre-immobilization, post-immobilization, and post-recovery) was conducted to test whether the placebo and creatine group responded differently throughout the whole study period. For muscle fiber characteristics, a second within-subjects factor was added (type I vs. II fibers).

The effects of the creatine-loading phase on muscle free creatine, phosphocreatine, and total creatine, as well as body weight, were tested using a two-way repeated measures ANOVA with time (all time points; and baseline vs. pre-immobilization separately) as within-subjects factor and treatment (creatine and placebo group) as between-subjects factors.

In case of a significant time × treatment interaction, paired *t* tests were performed in the creatine and placebo groups separately, with Bonferroni correction when appropriate (i.e., more than two time points). Data were analyzed using SPSS version 21 (SPSS, IBM Corp., Armonk, NY, USA). Results were considered statistically significant where *p* ≤ 0.05.

## Results

### Subjects

Baseline characteristics did not differ between the placebo and creatine groups (quadriceps CSA 7712 ± 324 and 8194 ± 263 mm^2^; *p* = 0.26; and leg muscle strength 56 ± 4 and 59 ± 3 kg; *p* = 0.47, respectively) (Table 1). Three subjects withdrew prior to immobilization due to time constraints. Of the 30 participants, 27 completed the study (placebo: *n* = 13; creatine: *n* = 14).

**Table 1:** Baseline characteristics

Characteristic	Placebo group (*n* = 13)	Creatine group (*n* = 14)
Age (years)	23 ± 1	23 ± 1
Body weight (kg)	73 ± 3	75 ± 2
Height (m)	1.76 ± 0.03	1.81 ± 0.02
BMI (kg/m^−2^)	23.5 ± 0.9	23.0 ± 0.5
1-RM (kg)	55 ± 4	59 ± 3
Quadriceps CSA (mm^2^)	7712 ± 324	8194 ± 263

Data are expressed as the mean ± standard error of the mean. No differences were found between groups (*p* > 0.05)

*1-RM* one-repetition maximum, *BMI* body mass index (kg·m^−2^), *CSA* cross-sectional area

### Creatine Loading

Muscle tissue free creatine, phosphocreatine, and total creatine content are displayed in Table 2. At baseline, muscle total creatine content averaged 89 ± 5 mmol/kg of dry muscle^−1^ (mmol/kg^−1^), with no differences between the placebo and creatine groups (88 ± 4 vs. 90 ± 9 mmol/kg^−1^, respectively; *p* = 0.68). Despite similar baseline values, an overall treatment effect was observed when all time points were included in the analysis, showing that muscle creatine content was greater in the creatine than in the placebo group (*p* = 0.026). When focusing on the loading phase, a significant main effect of time (*p* = 0.049) was observed, showing an overall increase in total creatine content. Although the time × group interaction did not reach statistical significance (*p* = 0.066), this increase in muscle total creatine content appeared to be driven by an increase in the creatine group (from 90 ± 9 to 107 ± 4 mmol/kg^−1^ following 5 days of loading), with no apparent changes in muscle total creatine contents in the placebo group (from 88 ± 4 to 90 ± 3 mmol/kg^−1^). In accordance, a significant time × treatment interaction was observed for muscle free creatine content (*p* = 0.011), with post hoc analyses showing a significant increase in the creatine group (*p* < 0.001) with no changes over time in the placebo group (Table 2). Correction of the raw data for ATP contents resulted in higher muscle total creatine content at baseline (103 ± 3 vs. 108 ± 5 mmol/kg^−1^ in the placebo vs. creatine group; *p* = 0.33), but the pattern of changes over time was in agreement with the raw data (Table 2). An overall effect of treatment was observed, with greater creatine contents in the creatine than in the placebo group (Table 2; *p* = 0.032). A main time effect was shown for the loading phase (*p* = 0.004), with no significant time × treatment interaction (*p* = 0.185).

**Table 2:** Muscle creatine content

	Placebo group (*n* = 13)				Creatine group (*n* = 13)			
	Baseline	Pre-immobilization	Post-immobilization	Recovery	Baseline	Pre-immobilization	Post-immobilization	Recovery
Free Cr	40 ± 2	44 ± 4	48 ± 4	40 ± 3	34 ± 1	48 ± 4^a^	53 ± 3^a^	52 ± 3^a^
CrP	48 ± 4	45 ± 4	40 ± 4	55 ± 5	55 ± 10	59 ± 6	45 ± 5	52 ± 5
Total Cr	88 ± 4	90 ± 3	87 ± 3	95 ± 4	90 ± 9^b^	107 ± 4^b^	98 ± 6^b^	104 ± 4^b^
Corrected total Cr^c^	103 ± 3	107 ± 4	104 ± 4	109 ± 3	108 ± 5^b^	119 ± 4^b^	128 ± 6^b^	124 ± 8^b^

Data are expressed as mean ± standard error of the mean. Free creatine, creatine phosphate, and total creatine content are expressed in mmol/kg of dry muscle^−1^

*ANOVA* analysis of variance, *Cr* creatine, *CrP* creatine phosphate

^a^Independent *t* tests showed no differences between treatments at baseline. Repeated measures ANOVA (time × treatment) showed significant time × treatment interaction (*p* = 0.01) for free creatine; Bonferroni corrected post hoc showed a significant increase compared with baseline in the creatine group only (all *p* ≤ 0.01)

^b^Independent *t* tests showed no differences between treatments at baseline. Repeated measures ANOVA (time × treatment) showed significant treatment effect (*p* = 0.03) for total creatine

^c^Total creatine contents corrected for the highest adenosine triphosphate content measured on the four time points of each individual

**Fig. 2:**
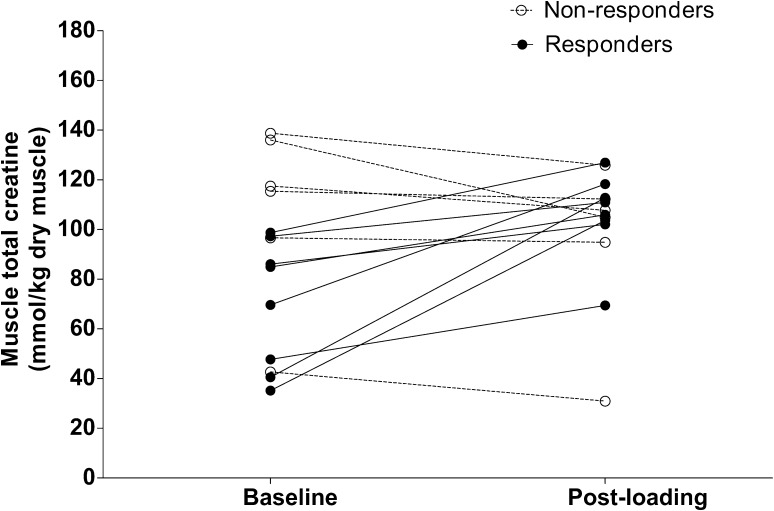
Individual changes in muscle total creatine at baseline and after 5 days of creatine loading in the creatine group. Responders to creatine supplementation (*n* = 8) were defined as subjects who showed an increase in muscle total creatine exceeding 10 mmol/kg^−1^ [10]. Non-responders (*n* = 6) were defined as subjects who showed no increase in muscle total creatine content or an increase <10 mmol/kg^−1^ creatine [10]

In the creatine group (*n* = 14), six subjects did not show an increase in muscle total creatine content exceeding 10 mmol/kg^−1^ and could therefore be considered non-responders to creatine loading [10]. In agreement with previous findings [10, 33], most of these non-responders showed higher baseline muscle creatine contents than did the responders (121 ± 8 vs. 70 ± 9 mmol/kg^−1^; *p* < 0.01). The individual responses to creatine loading are presented in Fig. 2. In the responders to creatine loading, we observed a significant increase in total creatine content in muscle from 70 ± 9 to 106 ± 6 mmol/kg^−1^ (*p* < 0.01). When correcting these raw data for ATP contents, a significant increase was observed from 102 ± 5 to 119 ± 6 mmol/kg^−1^ (*p* < 0.01).

**Fig. 3:**
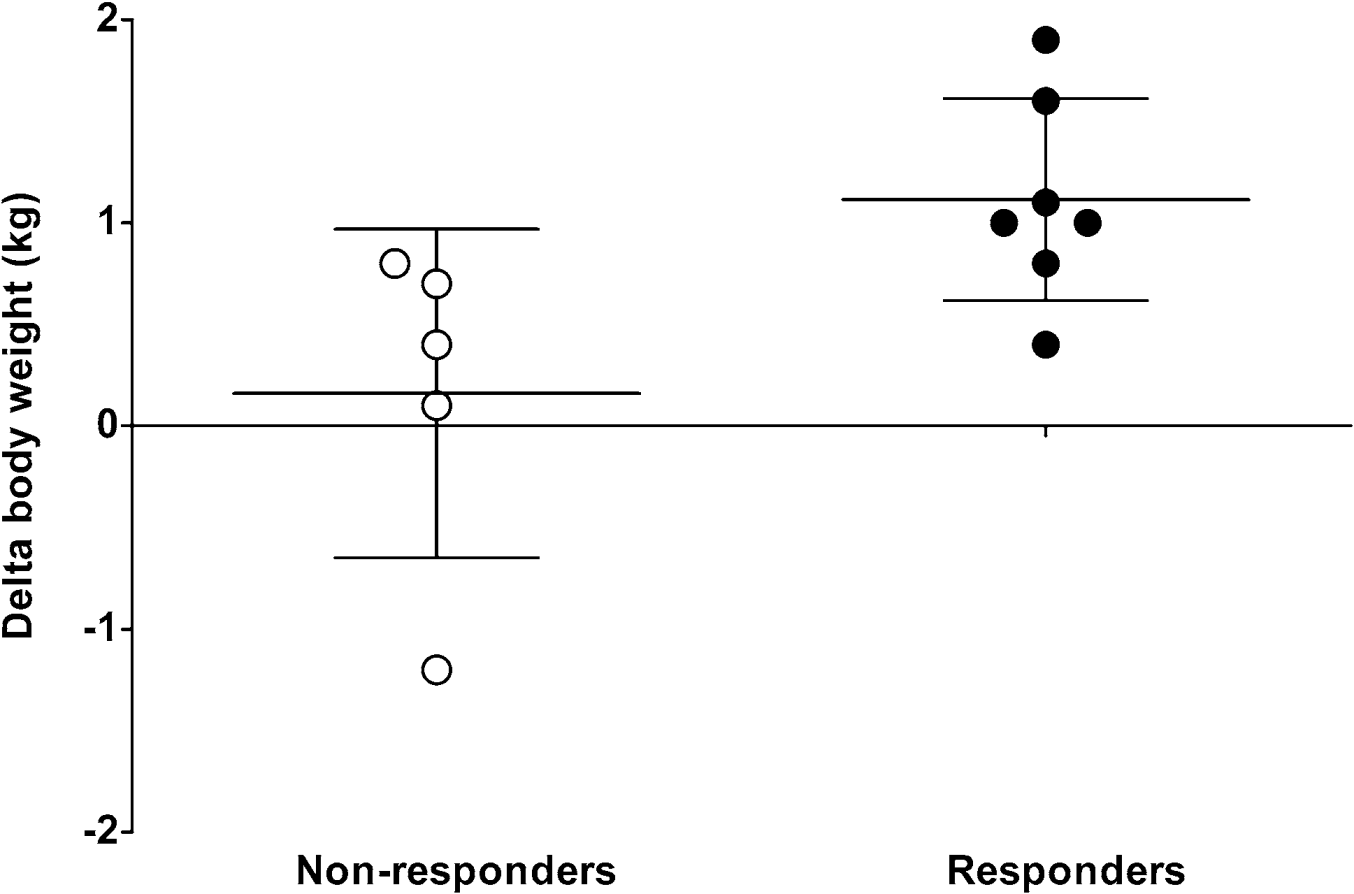
Individual changes in body weight in the responders and non-responders following 5 days of creatine loading. Body weight changes are calculated as post-loading minus baseline values

During the loading phase, body weight increased more in the creatine than in the placebo group (time × treatment interaction *p* = 0.02). Body weight increased from 73.7 ± 2.2 to 74.4 ± 2.3 kg in the creatine group (*p* = 0.01); no changes were observed in the placebo group (71.2 ± 2.8 to 71.4 ± 2.8 kg; *p* = 0.39). Within the creatine group, greater increases in body weight were observed in the responders to creatine loading than in the non-responders (*p* = 0.03). Individual changes in body weight in the responders and non-responders in the creatine group are presented in Fig. 3.

### Muscle Mass

**Fig. 4:**
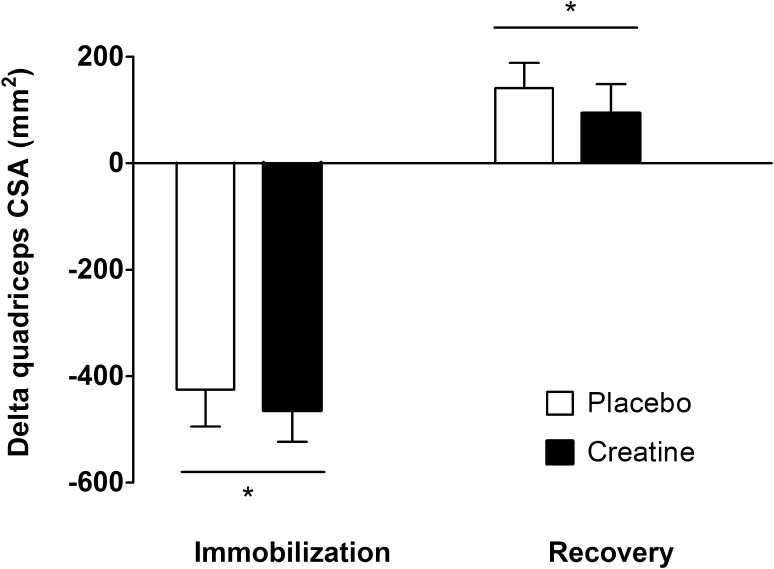
Changes in cross-sectional area of the quadriceps muscle in the creatine and placebo group following 7 days of one-legged knee immobilization and 7 days of subsequent recovery. Data were analyzed by two-way repeated measures analysis of variance, with time as a within-subjects factor and treatment (creatine and placebo group) as between-subjects factors. Data are expressed as mean ± standard error of the mean. Immobilization resulted in a significant decline in quadriceps cross-sectional area in both groups (*asterisk*), with no differences between groups. Quadriceps cross-sectional area increased during the recovery week (*asterisk*), with no differences between groups. *CSA* cross-sectional area

Leg immobilization resulted in a significant decline in muscle CSA of the quadriceps muscle (*p* < 0.01), with no differences between groups (Fig. 4; time × treatment interaction *p* = 0.76). Quadriceps CSA decreased by 5.5 ± 0.8% (from 7712 ± 324 to 7287 ± 305 mm^2^) in the placebo group. In the creatine group, quadriceps CSA decreased by 5.6 ± 0.8% (from 8194 ± 263 to 7729 ± 245 mm^2^). During the subsequent recovery phase, quadriceps CSA increased by 2.0 ± 0.6 and 1.1 ± 0.7% in the placebo and creatine group, respectively (*p* < 0.01), with no differences between groups (time × treatment interaction *p* = 0.52).

In agreement with the changes in quadriceps CSA, the CSA of the whole thigh muscle decreased during immobilization in both the placebo (3.6 ± 0.7%) and the creatine (3.7 ± 0.6%) groups, with no differences between groups (*p* = 0.76). During the recovery week, whole thigh muscle CSA of the total group increased by 1.2 ± 0.4% (*p* < 0.01), with no differences between groups (time × treatment interaction *p* = 0.52).

In the non-immobilized leg, quadriceps muscle CSA did not change differently in the placebo or creatine group during immobilization (respectively, –8 ± 33 and 41 ± 48 mm^2^; time × treatment interaction *p* = 0.41) or subsequent recovery (respectively, +62 ± 44 and +94 ± 47 mm^2^; time × treatment interaction *p* = 0.63). The responders to creatine loading showed a change in quadriceps muscle CSA that was similar to that in the non-responders to creatine loading during immobilization (respectively, –446 ± 88 and –489 ± 79 mm^2^) or recovery (respectively, +85 ± 41 and +106 ± 122 mm^2^).

### Knee Extension Strength

**Fig. 5:**
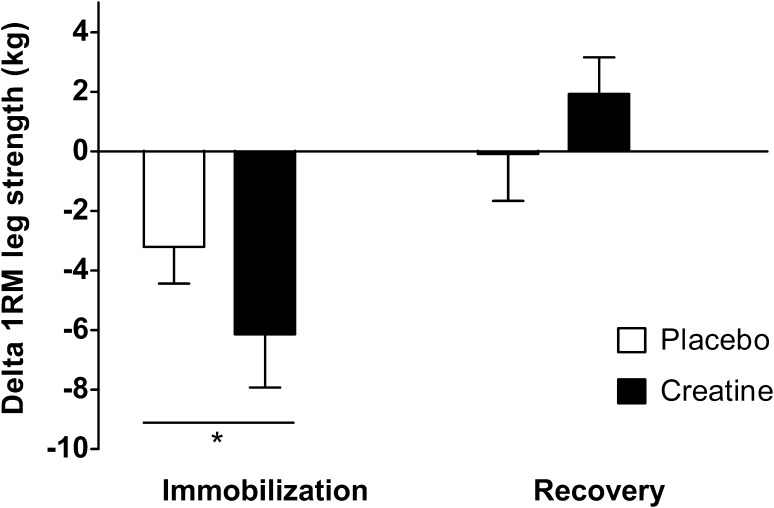
Changes in the one-repetition maximum as an estimate of leg muscle strength in the creatine and placebo group, following 7 days of one-legged knee immobilization and 7 days of subsequent recovery. Data were analyzed by two-way repeated measures ANOVA with time as a within-subjects factor and treatment (creatine and placebo group) as between-subjects factors. Data are expressed as means ± standard error of the mean. Immobilization resulted in a significant decline in muscle strength in both groups (*asterisk*), with no differences between groups. Muscle strength did not change during the recovery week, with no differences between groups. *1-RM* one-repetition maximum

1-RM decreased significantly during leg immobilization (*p* < 0.01), with no differences between groups (Fig. 5; time × treatment interaction *p* = 0.20). 1-RM decreased by 5.6 ± 2.2% (from 56 ± 4 to 53 ± 4 kg) in the placebo group and by 9.2 ± 3.1% (from 59 ± 3 to 53 ± 3 kg) in the creatine group. During the subsequent recovery week, 1-RM did not increase in either group, and no differences were found between the two groups (time × treatment interaction *p* = 0.32). The responders and non-responders to creatine loading showed a similar change in 1-RM during immobilization (respectively, –5 ± 3 and –8 ± 2 kg) and recovery (respectively, +1 ± 2 and +3 ± 1 kg).

1-RM in the non-immobilized leg increased during the immobilization period by 3.0 ± 1.5% (from 61 ± 2 to 63 ± 2 kg; *p* = 0.03), with no differences between the placebo and creatine groups (time × treatment interaction *p* = 0.90). 1-RM in the non-immobilized leg did not change during the subsequent recovery week (*p* = 0.57).

### Muscle Fiber Characteristics

Muscle fiber characteristics are presented in Table 3. At baseline, type I and II muscle fiber size averaged 6458 ± 334 and 7263 ± 375 μm^2^, respectively (*p* = 0.01), with no differences between groups. No significant changes in type I or II muscle fiber size were observed following leg immobilization or during subsequent recovery. The proportion of type I and type II muscle fibers was 39 ± 3 and 61 ± 3%, respectively, before the loading period. No changes in muscle fiber type composition were observed over time or between groups. No differences were observed in fiber size between responders and non-responders to creatine loading, and no changes were observed over time in either group.

**Table 3:** Muscle fiber characteristics in the placebo and creatine group

Characteristic	Fiber type	Placebo group (*n* = 13)				Creatine group (*n* = 13)			
		Baseline	Pre-immobilization	Post-immobilization	Recovery	Baseline	Pre-immobilization	Post-immobilization	Recovery
Muscle fiber CSA (μm^2^)	I	5991 ± 391	6034 ± 501	6620 ± 508	6078 ± 406	6965 ± 521	6898 ± 502	6935 ± 607	6813 ± 408
	II^a^	6671 ± 355	7202 ± 640	7540 ± 587	7028 ± 469	7855 ± 633	7434 ± 456	7630 ± 865	7471 ± 534
Fiber type distribution (%)	I	35 ± 3	38 ± 4	33 ± 3	39 ± 4	42 ± 4	46 ± 3	38 ± 5	46 ± 4
	II^a^	65 ± 3	62 ± 4	67 ± 3	61 ± 4	58 ± 4	54 ± 3	62 ± 5	54 ± 4
Fiber type distribution (% area)	I	33 ± 3	34 ± 4	31 ± 3	36 ± 4	40 ± 5	44 ± 3	37 ± 5	44 ± 5
	II^a^	67 ± 3	66 ± 4	69 ± 3	64 ± 4	60 ± 5	56 ± 3	63 ± 5	56 ± 5

Values are mean ± standard error of the mean. No differences were found between the placebo and creatine group for all variables on all time points

*CSA* cross-sectional area

^a^Muscle fiber CSA and fiber type distribution (in % and % area) differed between type I and type II muscle fibers on all time points

### Diet and Physical Activity

Baseline daily energy intake was 8.2 ± 0.4 MJ per day, with no differences between groups (*p* = 0.26). Dietary protein intake averaged 1.1 ± 0.1 g/kg^−1^ body weight per day, with no differences between groups (*p* = 0.61). Neither daily energy nor protein intake changed significantly during the subsequent immobilization and recovery period.

## Discussion

The present study shows that 7 days of one-legged knee immobilization results in a substantial decrease in muscle mass and strength in healthy young men. Creatine loading prior to immobilization and creatine supplementation during immobilization did not attenuate the loss of muscle mass or strength during immobilization. In addition, creatine supplementation failed to accelerate muscle mass or strength regain during 1 week of subsequent recovery.

Short-term disuse leads to substantial losses in muscle mass and strength. The current study demonstrates that merely 7 days of disuse leads to 5.5 ± 0.5% loss of muscle mass (Fig. 4), representing 0.8% loss of muscle mass per day. This muscle loss is in line with earlier work [1, 32, 34] and underlines the impact of even short periods of disuse. Besides the loss of muscle mass, muscle strength, as estimated by 1-RM, declined by 7.6 ± 2.0% (Fig. 5). Although maximal leg strength measurements may have been compromised by the muscle biopsy collection 1 h prior to strength testing, we observed declines in muscle strength (1–2% per day) similar to those reported previously in our laboratory as well as by others following a short period of limb immobilization [1, 32, 34]. The loss of muscle mass and strength during disuse is associated with a longer recovery period, a higher risk of injury reoccurrence, and a decline in metabolic health [3–6]. Hence, short-term disuse atrophy is of high clinical relevance, and effective strategies are needed to preserve muscle mass and strength during short periods of disuse or bed rest due to injury or hospitalization [35].

We hypothesized that creatine loading prior to disuse, thereby increasing muscle creatine content, attenuates the loss of muscle mass and strength. Therefore, the creatine group consumed 20 g creatine per day during the 5-day loading phase, which allowed total creatine content in muscle to increase by 19%. This loading and maintenance supplementation scheme has previously been shown to increase muscle free creatine and creatine phosphate [9, 11] and is in accordance with the guidelines of the American College of Sports Medicine [29]. In the present study, eight subjects showed an increase in total creatine content of >20%. In the creatine supplemented group, six subjects did not show a substantial increase in muscle creatine content (Fig. 2). Subjects not showing differences in muscle creatine content exceeding 10 mmol/kg^−1^ were defined as non-responders [10]. It has been well established that 15–30% of people who take creatine supplements do not experience increases in muscle total creatine contents [9, 10, 33]. Such non-responders to creatine supplementation typically show high(er) baseline muscle creatine contents, potentially preventing further increases in muscle creatine content [9, 10, 33]. In agreement, our non-responders showed substantially greater baseline creatine contents than responders (108 ± 14 vs. 70 ± 9 mmol/kg^−1^, respectively; *p* = 0.04). We also observed an increase in body weight in the responders to creatine supplementation, ranging from 0.4 to 1.9 kg, which is in line with findings of previous studies [9, 10, 29]. The increase in body weight with creatine loading has been found to result from water retention due to the increased cellular osmolarity [11, 17] or the increase in fat-free mass [9, 15, 16]. The fact that the individuals with an increase in muscle total creatine content also show an increase in body weight supports that these subjects are responders to creatine supplementation.

Despite the effective creatine loading, the greater muscle creatine content did not result in the preservation of muscle mass or strength during 7 days of single-leg immobilization. Findings were similar when only the responders to creatine supplementation were included in the analyses. In addition to the changes in muscle quadriceps CSA, we also assessed potential disuse atrophy on a muscle fiber level in the vastus lateralis muscle. Average type I and type II muscle fiber CSA showed no net changes following immobilization in both the placebo and creatine group (Table 3). The absence of a detectable decline in muscle fiber size following short-term immobilization has been reported previously [2] and is attributed to the substantial inter- and intra-subject variation in muscle fiber sizes [36]. Of course, the vastus lateralis muscle does not per se represent the entire quadriceps muscle. Furthermore, a muscle biopsy sample taken from the mid region of the vastus lateralis does not necessarily represent the entire muscle. Therefore, we can only assume that the presented data extend to all upper and/or lower leg muscles.

Though the current study focused on the impact of creatine loading on the decline in muscle mass and strength during immobilization, we also assessed changes in muscle mass and leg strength during the acute stages of recovery from disuse. We observed a 1.5 ± 0.5% regain in skeletal muscle mass after 1 week of recovery. Although significant, this increase in muscle mass represents only 26% of the muscle mass that was lost during the 7-day immobilization period. Thus, to regain all muscle mass after a period of disuse, the time required for rehabilitation will be much longer than the duration of the disuse period itself. Interestingly, no detectable regain in leg strength was observed after the first week of recovery. Creatine supplementation did not affect muscle mass or leg strength regain. Hespel et al. [21] previously showed greater muscle regain following a period of disuse when creatine was supplemented during a more prolonged period of rehabilitation including a 10-week resistance-type exercise training program. Clearly, although we show that creatine supplementation does not preserve muscle mass during disuse or support muscle regain during the acute stages of recovery from disuse, it may still be of benefit in support of muscle mass and strength regain during more prolonged active rehabilitation.

We hypothesized that creatine loading prior to and during immobilization would prevent or attenuate muscle loss during a short period of immobilization. The increase in muscle creatine content following creatine loading creates an osmotic potential that forces water in the muscle, causing muscle cells to swell [18]. Increases in cell volume and the impact on cell volume homeostasis have been associated with muscle anabolism [18]. Despite the successful creatine loading prior to the immobilization period, the greater muscle creatine content did not prevent or attenuate the decline in muscle mass or strength that is typically observed during short-term disuse. Therefore, in spite of frequent speculation on the clinical advantage of creatine supplementation during disuse, we have to remove creatine from the list of potential nutritional or pharmaceutical compounds that may help to prevent disuse atrophy. Of course, creatine supplementation can still provide an ergogenic advantage in anaerobic energy provision required during exhaustive intermittent-type exercise. As such, creatine supplementation may be used during clinical rehabilitation to augment the skeletal muscle adaptive response to prolonged resistance-type exercise training. However, it should be noted that muscle loss during disuse occurs at a rate that is several-fold greater than muscle (re)gain during resistance-type exercise training. Therefore, it is imperative that we continue our endeavor to identify nutritional or pharmaceutical compounds that may prevent or attenuate disuse atrophy [35].

## Conclusion

Creatine supplementation prior to and during leg immobilization does not prevent or attenuate the loss of muscle mass or strength during short-term muscle disuse in males.
